# HURT (Headache Under-Response to Treatment) questionnaire in the management of primary headache disorders: reliability, validity and clinical utility of the Arabic version

**DOI:** 10.1186/1129-2377-14-16

**Published:** 2013-02-21

**Authors:** Mohammed Al Jumah, Ali Al Khathaami, Hani Tamim, Abdulla Al Owayed, Suleiman Kojan, Ayah Jawhary, Richard Lipton, Dawn Buse, Richard Jensen, Timothy Steiner

**Affiliations:** 1 King Abdullah International Medical Research Center, King Saud Bin Abdulaziz University for Health Sciences, NGHA, KAIMRC, KSAU-HS, P.O. Box 22490, 11426, Riyadh, Saudi Arabia; 2 Department of Neurology, Albert Einstein College of Medicine and Montefiore Medical Center, Bronx, NY, US; 3 Danish Headache Center, Department of Neurology, Glostrup Hospital, University of Copenhagen, Copenhagen, Denmark; 4 Department of Neuroscience, Norwegian University of Science and Technology, Trondheim, Norway; 5 Division of Neuroscience, Imperial College London, London, UK

**Keywords:** Headache, Management, HURT questionnaire, Primary care, Reliability, Validation, Global campaign against headache

## Abstract

**Background:**

To support better headache management in primary care, the Global Campaign against Headache developed an 8-question outcome measure, the Headache Under-Response to Treatment (HURT) questionnaire. HURT was designed by an expert consensus group with patient-input. It assesses the need for and response to treatment, and provides guidance on actions to optimize therapy. It has proven content validity.

We aim to evaluate the Arabic version of HURT for clinical utility in primary care in Saudi Arabia.

**Methods:**

HURT was translated according to the Global Campaign’s translation protocol. We assessed test-retest reliability in consecutive patients of four primary-care centres, who completed HURT at two visits 4-6 weeks apart while receiving usual care. We then provided training in headache management to the GPs practising in these centres, which were randomized in pairs to control (standard care) or intervention (care guided by implementation of HURT). We assessed responsiveness of HURT to clinical change by comparing base-line responses to HURT questions 1-6 with those at follow up. We assessed clinical utility by comparing outcomes between control and intervention pairs after 3 months, using locally-developed 5-point verbal-rating scales: the patient-satisfaction scale (PSS) and doctor-satisfaction scale (DSS).

**Results:**

For test-retest reliability in 40 patients, intra-class correlation coefficients were 0.66-0.78 for questions 1-4 and 0.90-0.93 for questions 5-7 (all P ≤ 0.001). For the dichotomous response to question 8, Kappa coefficient = 1 (P < 0.0001). Internal consistency was good (Cronbach’s alpha = 0.74). In 342 patients, HURT signalled clinical improvement over 3 months through statistically significant changes in responses to questions 1-6. PSS scores were higher among those in whom HURT recorded improvement, and also higher among those with less severe headache at baseline. Patients treated with guidance from HURT (n = 207) were more satisfied than controls (n = 135), but this did not quite reach statistical significance (P = 0.06).

**Conclusion:**

The Arabic HURT Questionnaire is reliable and responsive to clinical change in Arabic-speaking headache patients in primary care. HURT showed clinical utility in this first assessment, conducted in parallel with studies elsewhere in other languages, but this needs further study. Other Arabic instruments are not available as standards for comparison.

## Background

It is well established that headache causes substantial disability worldwide [1] and is among the most commonly reported neurological disorders in primary care [2]. Because of their high prevalence and their disabling nature, tension-type headache (TTH), migraine and medication-overuse headache (MOH) are three disorders collectively responsible for the majority of headache-attributed burden [3-7].

In collaboration with the World Health Organization (WHO), the Global Campaign against Headache was launched by the non-governmental organization *Lifting The Burden* (LTB) in 2004 [8,9]. It has made progress since towards its objective of reducing the burden of headache worldwide [10]. LTB expressly recognizes that, because of the numbers of people affected, most headache disorders need to be managed in primary care [8], and accordingly has produced a range of management aids for use by non-specialist health-care providers (HCPs) [11]. These include an outcome measure, the HURT (Headache Under-Response to Treatment) questionnaire [12-14], an 8-item, self-administered questionnaire addressing headache frequency, disability, medication use and effect, perception of headache “control” and knowledge of diagnosis (see Additional file 1). The original instrument was created in English by an expert consensus group drawn from all six world regions, and including patients’ representatives, and refined through a multi-stage process consisting of item development, item reduction using item-response theory, and psychometric testing [12,13]. The first seven questions each have five categorical response options, graded from good to bad. Question 8 is dichotomous (yes/no). Responses are numerically coded, and can be summed, but questions address heterogeneous concepts related to care and outcome and provide greater information when analyzed separately. Specifically, while being designed as an outcome measure, HURT aims to guide management not only by indicating when treatment is or is not optimal but also by suggesting how management should be modified to improve outcome. This feature links the assessment to clinical advice and decision making in a way expected to be of particular help in primary care. Specifically how this is achieved can be seen from the instructions attached to HURT (Additional file 1).

In its original English version, HURT has been shown to be reliable, to function similarly across different headache disorders and to correlate well and in the expected directions with other validated measures (*e.g*., The Migraine Disability Assessment [MIDAS] questionnaire, the Headache Impact Test [HIT-6], the depression scale of the Patient Health Questionnaire [PHQ-9], health-related quality of life measure [HRQoL v2] and the Migraine Prevention Questionnaire [MPQ]) [12-14]. Psychometric validation of HURT is continuing. However, the purpose of drawing the formulating group from all world regions was to develop an instrument useful not only for all primary headache disorders but also cross-culturally. Test-retest reliability has been evaluated in headache specialist centres in Denmark, Italy and the United Kingdom [15], and assessments of clinical utility are being undertaken in multiple languages and countries. This study begins these processes for an Arabic version of HURT: more than 320 million people, and possibly 60 million with troublesome headache, are native Arabic speakers. We asked whether the Arabic version of HURT is reliable, responsive as an outcome measure in clinical practice and helpful to management by primary-care physicians (PCPs).

We were constrained by a lack of alternative instruments validated in the Arabic language or Saudi Arabian population that we might use as standards. To assess outcome, we applied simple locally-developed satisfaction scores.

## Methods

This prospective study was carried out in two stages in Riyadh City, Kingdom of Saudi Arabia.

### Ethics approval

The Institutional Review Board of National Guard Health Affairs, Saudi Arabia, approved the study.

### Translation

Translation into Arabic followed the very rigorous translation protocol developed by LTB [16] to achieve linguistic and conceptual equivalence between the Arabic and original English versions. In summary, two independent forward-translations by two Arabic native speakers fluent in English, one a headache expert (MJ), were reconciled through a translation coordinator. Back-translation was carried out by another bilingual headache expert, and the product compared with the original for equivalence, with further reconciliation as needed. A lay native Arabic speaker assessed the agreed translation for comprehensibility, and finally this was tested on 10 headache patients. Full details of these procedures are published elsewhere [16].

### Study participants

The study was conducted in four primary-care centres serving Saudi National Guard employees and their families in the city of Riyadh, which is reasonably representative of the Saudi population: relatively young, genetically homogeneous and with almost equal gender representation. Each centre had its own staff, and PCPs did not cross-cover other centres. Consecutive patients visiting any of these centres were included if complaining of headache, diagnosed by their PCP as having migraine, TTH or MOH, older than 18 years, Arabic-speaking and giving informed consent. Patients with trigeminal autonomic cephalalgias, secondary headache other than MOH, dementia or major psychiatric disorder (all psychoses and major depression) were excluded. The treating PCPs were responsible for applying the study inclusion and exclusion criteria.

All PCPs from the four centres attended a one-day workshop on the diagnosis and management of headache disorders in order to reduce inter-physician variability in knowledge and practice. Diagnostic work up, therapeutic interventions and frequency of follow-up were then left to the discretion of the treating physicians.

### Study design

The study was conducted in two stages from January 2009 to June 2010. Stage one assessed test-retest reliability and internal consistency. Patients answered all eight questions of HURT at their first (baseline) visits, and again at their second visits 4-6 weeks later, meanwhile receiving usual care.

Stage two had two purposes: first to assess the responsiveness of HURT to clinical change (reflecting its utility as an outcome measure) and second to examine its clinical utility in guiding PCPs’ management of headache disorders. After a review of their patterns of headache presentation and the numbers of PCPs in each, the four centres were paired so as best to eliminate differences between the pairs. Each had almost 20 PCPs. The two pairs were then randomly assigned to either intervention (PCPs using HURT to guide their management of patients) or control (PCPs continuing their usual practice). Each centre recruited patients during the following three months, with the patients of these pairs of centres in the intervention and control groups respectively. After six months, the control centres introduced the HURT Questionnaire into their practice, and their subsequent patients were added to the intervention group.

### Outcome measurement

All patients were seen at baseline, when HURT was applied to those in the intervention group. Effectiveness of management was assessed after 3 months: patients in the intervention group again completed HURT, while all patients answered two questions in a locally-developed patient-satisfaction scale (PSS) addressing headache frequency and control, and its effect on life. The response options to each, in a 5-point verbal-rating scale, were: very satisfied, satisfied, neither satisfied nor dissatisfied, dissatisfied, very dissatisfied. All treating PCPs answered two questions in a similar doctor-satisfaction scale (DSS), with the same response options, addressing diagnosis and management/control. The verbal responses were given numerical scores from +2 (very satisfied) to -2 (very dissatisfied). It was assumed that higher scores on these scales reflected better outcomes and better clinical management.

HURT responsiveness to clinical change was assessed in the intervention group (ultimately all patients) by comparing patients’ responses to questions 1-6 at first visit with those at the follow-up visit.

### Statistical analyses

Data were analyzed using Statistical Analysis Software (SAS) version 9.0. Student’s *t*-test and the chi-squared test were used to compare means and proportions respectively. Logistic regression analysis was carried out to identify predictors of satisfaction. The model included age, gender, level of education, marital status and use of HURT. Results were expressed as odds ratios (ORs) with 95% confidence interval (CI).

The various questions of HURT address heterogeneous concepts related to care and outcome, so greater information is provided when each is analyzed separately. We used the numerical codes assigned by HURT to the five response options to each of questions 1-7 (from 1 [most favourable] to 5 [least favourable]), and treated these as continuous variables, which we summarized by means and standard deviations (SDs). Reliability and internal consistency in these questions were assessed by intra-class correlation coefficient and Cronbach’s alpha respectively. For question 8, the dichotomous (yes/no) response options were scored yes = 1 and no = 0, and reliability was assessed by Kappa coefficient.

PSS and DSS numerical scores (derived as above) were analyzed as continuous variables and summarized by means and SDs. In addition, they were dichotomized to “satisfied” (score >0) and “dissatisfied” (score ≤0).

We took patient satisfaction (or lack of it) as the standard indicator of good (or bad) outcome. After the follow-up visit, patients in the intervention group were divided into two categories, “improved” and “worsened”, on each individual HURT question 1-4 (these four questions reflecting headache frequency, headache-attributed disability, and medication use). A patient was categorized as worsened when the difference between visits (follow-up minus first) was ≤0 (*i.e.*, including no change), and otherwise (difference >0) as improved. For example, on HURT question 1 (“on how many days in the last month did you have a headache?”), a patient answering “3-5” at both first and follow-up visits would be considered as worsened, since no benefit was reported despite treatment, whereas he/she would be considered improved only when the answer to the same question on follow up was “1-2” or “0”. We then analysed PSS scores within each category.

In a second analysis, aimed at showing that PSS scores meaningfully reflected clinical outcome, we related these scores to baseline headache severity. HURT grades the responses to questions 1-4 into four categories of severity (see Additional file 1); we dichotomized these, for each question, into “severe headache” (either of the two highest-severity categories) or otherwise “mild-to-moderate headache”. We then assessed PSS scores in each of these groupings.

For analysis of clinical utility, HURT was used only as the intervention; outcome measurements in intervention and control groups relied on PSS and DSS.

## Results

A total of 342 patients (27% male) were recruited, with mean age 34.8 (±11) years. The first 40 patients participated in the assessment of test-retest reliability. Intra-class correlation coefficient for HURT questions 1-7 ranged from 0.66 to 0.93, with highly significant P-values in each case (Table 1). For question 8, kappa =1 (P < 0.0001). For internal consistency, Cronbach’s alpha = 0.74.

**Table 1:** Correlations between test and retest responses 4-6 weeks apart to HURT questions 1-8 (n = 40)

**HURT question**	**Intra-class correlation coefficient**	**P-value**
1. On how many days in the last month did you have a headache?	0.74	<0.0001
2. On how many days in the last three months did your headaches make it hard to work, study or carry out household work?	0.66	0.001
3. On how many days in the last three months did your headaches spoil or prevent your family, social or leisure activities?	0.78	<0.0001
4. On how many days in the last month did you take medication to relieve a headache?	0.67	<0.0001
5. When you take your headache medication, does one dose get rid of your headache and keep it away?	0.92	<0.0001
6. Do you feel in control of your headaches?	0.90	<0.0001
7. Do you avoid or delay taking your headache medication because you do not like its side-effects?	0.93	<0.0001
8. Do you feel you understand [your headache] diagnosis?	Kappa = 1.00	<0.0001

Responsiveness of HURT was assessed in all 342 patients. Responses to all questions but one showed statistically significant improvement at follow up; responses to question 7 (“Do you feel in control of your headaches?”) showed statistically significant worsening (Table 2). In a question-by-question analysis for questions 1-4, patients who improved according to HURT had higher PSS scores (more satisfied) (Table 3). Patients who had mild-to-moderate headache at baseline were more satisfied at the follow-up visit (Table 4).

**Table 2:** Responsiveness of HURT questionnaire to clinical management between first and follow-up visits 3 months apart (n = 342)

** HURT question**	**Response on scale 1-5 mean (SD)**		**P-value**
	**First visit**	**Follow-up visit**	
1	2.9 (1.0)	2.2 (0.3)	<0.0001
2	2.3 (1.0)	1.8 (0.7)	<0.0001
3	2.4 (1.1)	1.8 (0.7)	<0.0001
**Score (Q1 + Q2 + Q3)**	7.7 (2.6)	5.8 (2.0)	<0.0001
4	2.5 (1.1)	1.8 (0.8)	<0.0001
5	2.8 (1.2)	2.3 (0.9)	<0.0001
6	2.9 (1.1)	2.6 (0.9)	<0.0001
**Score (Q5 + 6)**	5.6 (2.0)	4.9 (1.6)	<0.0001
7	3.0 (1.3)	3.3 (1.1)	0.0007
8	**Patients responding “yes” n (%)**		
	82 (40.6%)	109 (54.0%)	<0.0001

Reductions indicate improvement, except for question 8.

**Table 3:** **Improvement as indicated by individual HURT questions (follow-up *vs *first visits) is associated with higher Patient Satisfaction Scores (PSS) at follow-up visit**

	**Mean PSS (SD)**		
** HURT question**	**Patients who improved**	**Patients who worsened**	**P-value**
**1**	0.91 (1.71) (n = 127)	-0.41 (2.16) (n = 79)	<0.0001
**2**	1.18 (1.74) (n = 92)	-0.20 (1.98) (n = 155)	<0.0001
**3**	1.22 (1.67) (n = 90)	-0.19 (2.02) (n = 115)	<0.0001
**4**	0.95 (1.81) (n = 107)	-0.16 (2.04) (n = 100)	<0.0001

**Table 4:** Baseline headache severity as measured by individual HURT questions is inversely related to Patient Satisfaction Scores (PSS) at follow-up

	**Mean PSS (SD)**		
**HURT question**	**Patients with mild-to-moderate headache at first visit**	**Patients with severe headache at first visit**	**P-value**
**1**	0.58 (1.91) (n = 191)	-1.73 (1.94) (n = 15)	<0.0001
**2**	0.70 (1.89) (n = 175)	-1.13 (1.91) (n = 32)	<0.0001
**3**	0.67 (1.90) (n = 177)	-1.07 (1.95) (n = 30)	<0.0001
**4**	0.72 (1.90) (n = 172)	-1.06 (1.83) (n = 35)	<0.0001

As for clinical utility of HURT, the demographics of the intervention (n = 207) and control (n = 135) groups showed small but significant mismatches in gender distribution and educational level (Table 5). Patients were more satisfied in the intervention group (*i.e.*, those in whom management was guided by HURT), but this did not quite reach statistical significance (0.52 *vs* 0.41; P = 0.06). There was no difference in DSS between PCPs who did and those who did not use HURT (0.77 *vs* 0.74; P = 0.57).

**Table 5:** Demographic characteristics of the control and the intervention groups

	**Control n = 135**	**Intervention n = 207**	**P-value**
**Age (years) (mean ± SD)**	34.8 ± 11.07	34.2 ± 12.3	0.60
**Male (n [%])**	48 [35.6]	43 [20.8]	0.003
**Married (n [%])**	99 [75.0]	162 [79.0]	0.39
**University education (n [%])**	23 [17.0]	15 [7.2]	0.005

Logistic regression analysis found female gender was the only significant predictor of patient satisfaction: women were more likely to be satisfied (OR = 2.0; 95% CI: 1.2-3.1; P = 0.003).

## Discussion

Our study was the first to translate and test the HURT Questionnaire in clinical use in an Arab population. It showed that HURT in Arabic language and applied to a population of Arabic-speaking headache patients in primary care is a reliable instrument. The 4-6-week period between test and retest balanced potential recollection bias (retest being influenced by the patients’ possible recollections of his or her previous responses) against the likelihood of real change in the disease during the test-retest interval. Questions 1-4 showed moderate but significant correlations (ranging from 0.66 to 0.78). These are acceptable, and at levels expected for this type of instrument, for questions that require recall of symptoms and medication use over the preceding 1-3 months [17,18]. For questions 5-7, excellent correlations were noted (ranging from 0.90 to 0.93) [17,18]. This reflects the more opinion-based nature of these questions and their relationship to present time rather than being recall-dependent. Internal consistency (Cronbach’s alpha = 0.74) was also acceptable.

We have also shown that HURT, in Arabic, is responsive as an outcome measure. Although the clinical change between baseline and follow-up visits was not quantified (no “gold-standard” measure exists), it was probably real for two reasons. First, most change was toward improvement, which must be expected after 3 months of medical treatment. Second, patients in whom HURT questions 1-4 signalled improvement reported satisfaction (positive PSS scores), while those in whom HURT signalled worsening (or no improvement) reported dissatisfaction (negative PSS scores). The opposite direction of change in the responses to question 7 was unexpected, but it might, perhaps, be explained. This question addresses patients’ feelings about headache control in general, and may have been interpreted in different ways. Some patients may have understood it to be asking about a “cure” for their condition, rather than effective management or control. It may well be that (some) patients’ expectations were unduly high and consequently unmet, or, very possibly, that 3 months was not sufficient to engender a feeling of control.

Validation of an outcome measure against expressions of patients’ satisfaction is methodologically debatable. We chose this approach for two reasons. First, there is no other outcome measure validated for Saudi Arabian culture. This was decisive on its own, but, second, patients’ satisfaction is of itself an important aspect of outcome. The drawback is that patients’ satisfaction has many determinants. It would be out-of-place here to discuss the large literature on this (none of it related to a Saudi population). However, while change in the disease itself is of course among these determinants, so, and importantly, is change in the way patients cope with and perceive their disease. The latter is highly subject to prior expectation, which may or may not be reasonable (either too high or too low). Nevertheless, the clear correlation, in the expected direction, between patients’ satisfaction and change as quantified by HURT strongly suggests that HURT detected and measured real change.

Whether change was due solely to standard care or improvement was enhanced by PCPs’ use of HURT is not absolutely clear: we found only a strong trend (P = 0.06) towards greater satisfaction in patients in the intervention (HURT) group compared with those in the control (standard care) group. Although the PSS was locally developed and itself not previously validated, we believe we showed here that PSS scores were an indicator, generally, of good outcome. But, for the reasons given above, patients’ satisfaction may be neither sensitive nor specific enough to reflect any effect of an intervention of this sort. DSS scores showed no difference between groups. The DSS was also locally developed and unvalidated. Doctors’ satisfaction has different determinants: it is likely of course to be increased by improved outcomes, but it may also be decreased by use of an outcome measure that indicates outcomes could be better (as HURT is intended to do). To establish the clinical utility of HURT as a management aid needs further study, but the lack of a gold-standard outcome measure (a gap that HURT was designed to fill) remains as an impediment to such study.

The study had one other limitation. For practical reasons, we randomized physicians rather than patients. Although all physicians received similar training, outcome differences between groups could in part have reflected differences in practice. Any such influence was partially offset by switching the two control centres to intervention, applying HURT, during the last six months of the study. Although this introduced the possibility of a period effect, it was unlikely that this was large or significant, and anyway it was diluted. We do not believe the minor differences between control and intervention groups in gender and level of education (Table 5) would have had significant impact on the comparison.

## Conclusion

The HURT Questionnaire in the Arabic language is a reliable and responsive outcome measure in an Arabic-speaking population of headache patients in primary care. It detects change in illness over time, but its clinical usefulness as an aid to management needs further study.

## Competing interests

All authors declare that they have no competing interests.

## Authors’ contributions

MJ and SK and AJ had carried out study data accurate. Whereas, MJ and AK and HT and AO and TJS had participated in the analysis and work plane, as for RBL and DCB and RJ and MJ, they handled part of study/ data plan and analysis. Finally all authors read and approved the final manuscript.

## Supplementary Material

Additional file 1 HURT Questionnaire. Click here for file
